# Characterization of a BAC transgenic mouse expressing *Krt19*-driven iCre recombinase in its digestive organs

**DOI:** 10.1371/journal.pone.0220818

**Published:** 2019-08-08

**Authors:** Tomohiro Kanayama, Hiroyuki Tomita, Nguyen Huy Binh, Yuichiro Hatano, Hitomi Aoki, Hideshi Okada, Akihiro Hirata, Yoshitaka Fujihara, Takahiro Kunisada, Akira Hara

**Affiliations:** 1 Department of Tumor Pathology, Gifu University Graduate School of Medicine, Gifu, Japan; 2 Physiology Department, Hanoi Medical University, Hanoi, Vietnam; 3 Department of Tissue and Organ Development, Gifu University Graduate School of Medicine, Gifu, Japan; 4 Department of Emergency and Disaster Medicine, Gifu University Graduate School of Medicine, Gifu, Japan; 5 Division of Animal Experiment, Life Science Research Center, Gifu University, Gifu, Japan; 6 Research Institute for Microbial Diseases, Osaka University, Suita, Osaka, Japan; Texas A&M University, UNITED STATES

## Abstract

Cytokeratin 19 (KRT19) protein is highly expressed in the epithelium of the gastrointestinal (GI) tract, hepatobiliary tissues, and pancreas of humans and mice. In the present study, we used an improved *Cre* (*iCre*) gene to enhance the efficiency of Cre expression in mammalian cells. We established a new transgenic *Krt19-iCre* bacterial artificial chromosome (BAC) mouse model using the BAC recombineering strategy. Site-specific iCre expression pattern was examined in embryos, adults, and elderly *Krt19-iCre* mice crossed with Tomato or LacZ reporter mice. Both iCre and reporter protein expressions in adult *Krt19-iCre;Tomato*^*flox/+*^ *(Krt19-iCre* Tomato reporter) mice were observed mainly in the epithelial cells of the GI tract, hepatobiliary tissues, and pancreas. However, the expression in the intrahepatic and small pancreatic duct were lower than those in the common bile and large pancreatic duct. In the *Krt19-iCre*; LacZ reporter embryos, β-galactosidase for the LacZ reporter was expressed in the glandular epithelial cells of the GI tract in 9.5-day embryos, 12-day embryos, and newborn mice. The reporter protein expression in *Krt19-iCre*-Tomato reporter mice was consistent with the KRT19 expression in human GI tissues. In conclusion, *Krt19-iCre* BAC transgenic mice can be used to investigate developmental and pathological conditions using the iCre-loxP system.

## Introduction

KRT19, an epithelial-specific typical type I keratin (~40 kDa) protein, is found in a broad range of epithelial tissues [1]. In mice, it is expressed in the epithelium of the stomach, small intestine, colon, pancreatic ducts of the adult pancreas, and the hepatobiliary ducts [2, 3]. In humans, KRT19 immunohistochemistry (IHC) is frequently used in diagnostic pathology to confirm the epithelial immunophenotype of undifferentiated tumors or to identify whether tumors of unknown primary origin are biliary, pancreatic, or renal ductular in origin [4, 5].

Cre/loxP-mediated DNA recombination allows for the functional analyses of essential genes in specific tissues. Thus, transgenic mice expressing Cre DNA recombinase in tissue-restricted patterns serve as practical tools to examine developmental and pathological conditions. A *Krt19-Cre* knock-in mouse line has been used to demonstrate that the mutated β-catenin gene induces intestinal polyposis [6]. Another Krt19-Cre mouse, in which Cre recombinase was inserted in the Krt19-promoter region, was crossed with a transgenic mouse strain with PTEN conditional alleles and PTEN protein expression was abrogated in the stomach and intestine [7]. Furthermore, *Krt19-CreERT* mice have demonstrated conditional DNA recombination in epithelial cells of multiple endodermal organs [8]. These reports demonstrate that *Cre*-mediated DNA recombination in Krt19-positive tissues is useful for studying normal developmental and pathological conditions. However, in mice, it is still unclear whether Krt19 is specifically expressed in the epithelium of the GI tract, hepatobiliary tissues, and pancreas; the human equivalents of which have been shown to be sites of KRT19 expression. Previous reports [6, 8] using *Krt19-Cre* mice have predominantly focused only on the stomach and parts of the intestine; therefore, a comprehensive comparison of Krt19 expression between mice and humans is warranted using *Cre*-mediated DNA recombinant mouse models.

The *Cre* gene comprises an undesirably high frequency of CpG dinucleotides, which can induce epigenetic silencing during mammalian development. Shimshek et al. [9] designed an improved *Cre* (*iCre*) gene, which is more efficiently expressed than the traditional one. Thus, several useful iCre transgenic mice lines have been recently generated [10–13].

In this study, we established transgenic *Krt19-iCre* mice using the bacterial artificial chromosome (BAC) recombineering strategy. On crossing with reporter mice, excellent iCre expression was observed in the progeny mice. This expression mimicked the KRT19 expression in the GI tract, hepatobiliary tissues, and pancreas of humans.

## Methods

### Ethics statement

This study was carried out in strict accordance with the recommendations in the Guide for the Care and Use of Laboratory Animals of the Gifu University. The protocol was approved by the Committee on the Ethics of Animal Experiments of Gifu University (30–21). All surgery was performed under isoflurane anesthesia, and all efforts were made to minimize suffering.

This use of human tissues in this study was approved by the Institutional Review Board of Gifu University and all human samples were obtained from Gifu University Hospital. Full written informed consent was obtained for all samples used in this study.

### Animal care

Animals were housed in a temperature-controlled room (22 ± 1°C) and constant humidity conditions on a 12-h light/dark cycle and were fed *ad libitum* in Gifu University Animal Facility. Euthanasia of experimental animals was performed by cervical dislocation after anesthesia with isoflurane.

### Generation of Krt19-iCre BAC clone and mice

**Fig 1:**
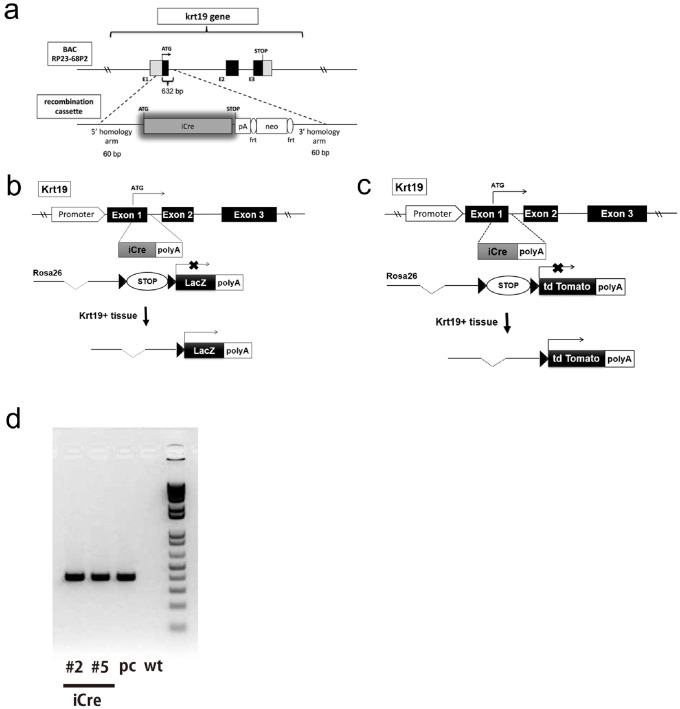
Generation of Krt19-iCre BAC transgenic mice. (**a**) Schematic representation of the *Krt19-iCre* construct. *Krt19-iCre* was constructed by inserting the *iCre* gene and polyA signal cassette into the coding region of the mouse *Krt19* locus. The cassette was recombined into the locus immediately after the start codon of exon 1. (**b**) Schematic representation of the *lacZ* reporter system. (**c**) Schematic representation of the *Tomato* reporter system. (**d**) PCR products were detected in #2 and #5 founders, the positive control (pc), and wild-type mice (wt). Genomic DNA was extracted from the tail of each mouse.

A BAC clone (BACPAC resources CHORI, RP24-68P2, 197 kb) containing the entire *Krt19* gene witha154-kb 5′ upstream sequence was used. Recombineering was performed according to protocols described previously [14–18]. An *iCre-polyA-FRT-neo-FRT* cassette was purchased from Genebridges (Heidelberg, Germany; http://www.genebridges.com/) and amplified with 60-bp homologous arms of the target site attached to each end by PCR. The sequence of the left arm was 5′-gcggccagcagttctcagacctgcgtccctttttccttcgctctggtctccctcctcatcATGGTCTCCAACCTGCTGAC (lowercase: 60 bp *Krt19* gene; uppercase: *iCre-polyA-FRT-neo-FRT* cassette start sequence), while that of the right arm was 5′- ctccccgccctgccgcaccaccccaacaggtgcacctcccagggcctggccgctacccacAATTAACCCTCACTAAAGGG (lowercase: 60 bp *Krt19* gene; uppercase: *iCre-polyA-FRT-neo-FRT* cassette end sequence). The PCR product was inserted into the RP24-68P2 BAC clone using homologous recombination in *E*. *coli* strain SW105 obtained from the Frederick National Laboratory for Cancer Research (Frederick, MD, USA)[14]. The ATG codon and the following 632 bps of the coding sequence in the *Krt19* gene were replaced with *iCre* cDNA (Fig 1a)[19]. Following removal of the neomycin (neo) selection marker by FLP-mediated recombination induced by L-arabinose, the concentration of the entire BAC fragment was adjusted to 1 ng/μL and was microinjected into the pronuclei of B6D2F1 mouse oocytes [20].

Twenty pups were produced, of which 12 contained one copy of the *Krt19-iCre* vector in their genome. The BAC DNA is randomly integrated into the mouse genome and can destroy the expression of other inherent mouse genes. However, the BAC transgene integrity has not always been elucidated in published studies [21]. Therefore, we used only heterogeneous mice, carefully checked for the endogenous Krt19 expression, observed the health condition at least for one year, and analyzed the macroscopic and microscopic findings of the founders.

Further, five founders had little or no LacZ expression when crossed with Rosa26-LSL-LacZ reporter mice. Three mice had no pregnancies and two mice had a low body weight, even as adults. Thus, we euthanized the 10 founders humanely.

Two of these 12 *Krt19-iCre* positive mice, Krt19-iCre#2 and Krt19-iCre#5, showed no health issues or major fertility defects and were chosen for the follow-up characterization of iCre expression. Thus, we used the Krt19-iCre#2 mouse line, with heterogeneous but no homogeneous alleles, in this study. All the experiments in this study were examined using two embryos, six adults, and two elderly mice and the expression patterns were the same in each mouse line. All mice used in this study remained healthy throughout the study.

### Reporter mice

*Tomato* ^*flox/flox*^ reporter mice (B6;129S6-*Gt(ROSA)26Sor* ^*tm9(CAG-tdTomato)Hze/J*^*)* [22] and *LacZ* ^*flox/flox*^ reporter mice (B6.129S4-*Gt(ROSA)26So r*^*tm1Sor/J*^) [23]were purchased from the Jackson Laboratory(Bar Harbor, ME, USA). To establish the reporter system using *lacZ* or *Tomato*, the *Krt19-iCre* mice were crossed with *Tomato* ^*flox/flox*^ or *LacZ* ^*flox/flox*^ reporter mice (Fig 1b and 1c).

In the embryological study, the day a vaginal plug was detected was designated embryonic day 0.5 (E0.5). The *Krt19-iCre*; *Tomato*^flox/+^ or *Krt19-iCre*; *LacZ* ^flox/+^ mice were sacrificed at 8–10 weeks of age, while pregnant females carrying *Krt19-iCre*; *Tomato*^flox/+^or *Krt19-iCre*; *LacZ* ^flox/+^ embryos were sacrificed at E9.5, E12, E17, and postnatal day 0 (P0).

### Genotyping

The *Krt19-iCre* transgenic mice were genotyped using the following primers: Krt19-5′UTR (forward primer), 5′-cagctctgggaaggactgag-3′ and iCre (reverse primer), 5′-gcatcttccaggtgtgttca-3′. The PCR profile was 30 cycles of denaturation, annealing, and elongation at 94 °C for 30 s, 55 °C for 30 s, and 72 °C for 30 s, respectively. The PCR products were predicted to be 398 bp in length(Fig 1d).

### Human tissue samples

Twelve human samples, which included normal tissues, were collected from nine patients who underwent surgical resection or biopsy at Gifu University Hospital. In each sample, normal tissue surrounding the lesions was identified by two certificated pathologists (T.K. and H.T.). More detailed case information is shown in S1 Table.

### Immunohistochemistry and immunofluorescence

The mice were perfused with 10% neutral buffered formalin and dissected. The adult and embryo tissues were fixed in 10% neutral buffered formalin overnight. The tissues were dehydrated with 70% ethanol and embedded in paraffin blocks. These blocks were cut into 3-μm thick sections. For IHC, the slides were deparaffinized in xylene and rehydrated using a graded alcohol series. The slides were heated in 10 mM citrate buffer (pH 6.0) at 120 °C for 1 min for antigen retrieval in a Pascal pressure chamber (DAKO, Glostrup, Denmark). Following immersion in 0.3% H_2_O_2_ in methanol to quench endogenous peroxidase activity, the slides were blocked with 2% bovine serum albumin for 40 minutes. Each section was incubated with a primary antibody(described below) at 4 °C overnight. The slides were then incubated with the secondary antibody (Vectastain Elite ABC Kit; Vector Laboratories, Burlingame, CA, USA) at room temperature for 30 min and subsequently treated with ABC reagent (Vectastain Elite ABC Kit) at room temperature for 30 minutes. The reaction products were visualized using ImmPACT DAB (Vector Laboratories). The slides were counterstained with hematoxylin.

To investigate the expression of human endogenous KRT19,anti-KRT19 antibody (rabbit monoclonal IgG, 1:800, Abcam, Cambridge, MA, USA) was used as a primary antibody. To determine the expression level of β-gal and iCre, anti-β-galactosidase (chicken polyclonal IgG, 1:1000, Abcam) antibody and anti-Cre antibody (1:200, Cell Signaling Technology, Danvers, MA, USA) were used as primary antibodies, respectively. Rabbit or chicken IgG were used as negative controls.

For immunofluorescence (IF), rabbit anti-RFP primary antibody (1:100, ROCK LAND, Gilbertsville, PA, USA) and anti-Krt19 antibody were used in a method similar to IHC. Rabbit IgG antibody was used for the negative control (1:100, DAKO, Japan). The slides were incubated with a secondary antibody (Goat polyclonal Secondary Antibody to Rabbit IgG, Alexa Fluor 594, pre-adsorbed, 1:200, Abcam and goat polyclonal Secondary Antibody to chicken IgG, Alexa Fluor 594, pre-adsorbed, 1:200, Abcam) at room temperature for 30 minutes. Then, the slides were incubated with DAPI solution (1:1000, WAKO, Japan) under light-shielded conditions at room temperature for 5 minutes. Finally, the slides were covered with cover slips.

### *In situ* hybridization and evaluation

We used RNAscope (Advanced Cell Diagnostics, Inc., California, USA) (Wanget al., 2012), an RNA *in situ* hybridization (ISH) method that can detect RNA in paraffin-embedded tissues. Three-μm section slides were deparaffinized in xylene and rehydrated using alcohols, then air-dried. The slides were incubated with an *iCre* probe (RNAscope Probe-*iCre*, #423321) or negative control probe (RNAscope Probe-*negative control*, #310043), and the signal was amplified using the RNAscope 2.0 HD Detection kit (Advanced Cell Diagnosis, Inc.) according to the manufacturer’s instructions and a previous report [24]. The expression was scored on a five-grade scale, as described in the manufacturer’s protocol (https://acdbio.com/services/quantitative-analysis). Briefly, the dots per five high-power fields were counted, the average number was calculated, and the score was defined as follows: 0, no staining or less than 1 dot/10 cells; 1+, 1–3 dots/cell; 2+, 4–9 dot/cell and very few dot clusters; 3+, 10–15 dots/cell and the clusters were less than 10% dots; 4+, >15 dots/cell and the clusters were more than 10% dots.

To confirm the co-localization of *iCre* mRNA expression and Krt19 protein expression, we performed the combined ISH and IHC technique according to a previous report [25]

#### Quantitative RT-PCR

RNA was extracted from P56 Krt19-iCre mice and wild-type mice using an RNeasy Mini kit (Qiagen, Tokyo, Japan). mRNA (500 ng) was reverse transcribed with SuperScript III (Invitrogen) at 55 °C for 1 hour into total complementary DNA (cDNA), which was used as the template for subsequent PCR reactions and analysis. The expression of *iCre* RNA was examined by real-time polymerase chain reaction (RT-PCR) using SYBR Green Detection Chemistry (Applied Biosystem). cDNA (500 ng) was used as the template for qPCR amplification in the presence of 10 μM specific primers, in a total volume of 20 μl. All reactions were run in triplicate and each reaction was also run without cDNA as negative controls. StepOne software v2.3 (Applied Biosystem) was used to convert the fluorescent data into cycle threshold (delta-Ct) measurements and the relative amount of specific transcript was calculated by the comparative cycle threshold method. All values of RNA accumulation of the specific genes were normalized to the signal of *β-actin*.

The primers for *iCre* and β-actin were designed as follows: *iCre* (forward tcctgtacctgcaagccaga, reverse catcaccagggacacagcat) and *β-actin* (forward gtgacgttgacatccgtaaaga, reverse gccggactcatcgtactcc).

### X-gal staining

To detect the localization of iCre expression in mouse embryos, we used *Krt19-iCre; lacZ* transgenic mouse embryos to detect β-gal activity by 5-bromo-4-chloro-3-indoxyl-beta-β-D-galactopyranoside (X-gal) staining. Briefly, E9.5 embryos were dissected away from the uterus and fixed 1 hour in 0.2% glutaraldehyde. After the embryos were washed 3 times with buffer for 15min, they were stained with X-gal (Roche, Indianapolis, IN, USA) for six hours under light-shielded conditions. Whole-mount-stained embryos were fixed in 10% neutral buffered formalin overnight. The tissues were dehydrated with 70% ethanol and embedded in paraffin blocks. These blocks were cut into 3-μm thick sections. After counterstaining using nuclear fast red stain, the slides were covered with cover slips.

## Results and discussion

### *iCre* mRNA expression in the GI tract of Krt19-iCre mice

**Fig 2:**
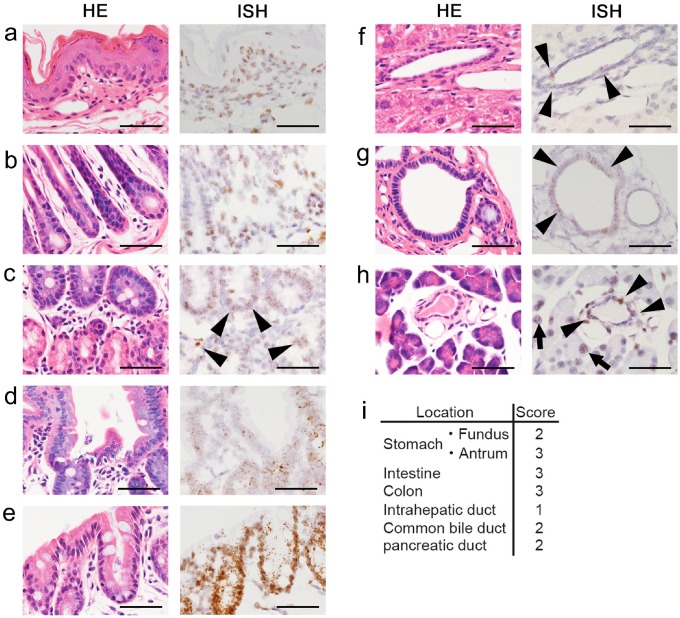
Expression patterns of *iCre* mRNA in the GI tract, hepatobiliary tissues, and the pancreas of adult Krt19-iCre mice. HE-staining and *in situ* hybridization of the epithelium of the esophagus (**a**), fundus and pylorus of the stomach (**b**), duodenum (**c**), small intestine (**d**), colon (**e**), cholangiocytes of the intrahepatic bile duct and common bile duct (**f** and **g**, respectively), and epithelial cells of the pancreatic duct (arrowheads) and in some exocrine cells(arrows) (**h**). Scale bars: 50μm. (**i**) Scores for the epithelial cells of the GI tract, hepatobiliary tissues, and the pancreas. The expression in the epithelial cells was scored on a five-grade scale as described in the manufacturer’s protocol (https://acdbio.com/services/quantitative-analysis) and was read as follows: 0, no staining or less than 1 dot/10 cells; 1+, 1–3 dots/cell; 2+, 4–9 dot/cell and very few dot clusters; 3+, 10–15 dots/cell and the clusters were less than 10% dots; 4+, >15 dots/cell and the clusters were more than 10% dots.

To investigate mRNA expression in the GI tract, hepatobiliary tissues, and the pancreas of *Krt19-iCre* mice, we performed *in situ* hybridization (ISH) using the RNAscope assay for three adult mice. *iCre* mRNA expression was detected in the epithelial cells of the entire GI tract, i.e., the esophagus, fundus and pylorus of the stomach, duodenum, ileum, jejunum, and colon (Fig 2a–2e). *iCre* mRNA expression was also observed in the epithelial cells of the biliary tree, including the common bile duct, intrahepatic bile duct, and pancreatic duct (Fig 2f–2h). These expression patterns were consistent among each tissue from the three mice. The expression of *iCre* mRNA was also confirmed by quantitative RT-PCR. While tissues from the gastrointestinal tract, hepatobiliary tract, and pancreas of the transgenic mice showed amplification of *iCre* mRNA, tissues from wild-type mice did not express *iCre* mRNA(S1 Fig). The expression patterns of *iCre* mRNA were consistent with the expression of endogenous Krt19 protein in the digestive organs, liver, and pancreas. However, in the small intestine, *iCre* mRNA expression in the three samples was found mainly in transient amplifying cells and it was restricted compared to the endogenous Krt19 expression (S2 Fig). Furthermore, some hepatocytes in the liver and the acinar exocrine cells in the pancreas showed *iCre* mRNA expression (Fig 2f and 2h). Scoring of the epithelial expression levels revealed that *iCre* mRNA expression in the intrahepatic bile duct was lower than that in the GI tract, the common bile duct, and the pancreatic duct (Fig 2i).

These data are consistent with those of a previous study [8] which demonstrated that in inducible Krt19-CreERT knock-in mice, the EYFP reporter protein expression level was lower in cells of the intercalated ducts, such as the intrahepatic and small pancreatic ducts, compared to that in cuboidal epithelial cells, such as the common bile duct and main pancreatic duct cells. Unfortunately, even though iCre recombinase was used instead of Cre recombinase, we were unable to improve low reporter protein expression levels in the intercalated ducts of the liver and pancreas.

In addition, there were signals in connective tissues of the esophagus, and these signals were specific because no hybridization signal was detected in other GI tract tissues using a negative control probe (Fig 2a and S2 Fig).

#### Reporter expression in the GI tract of Krt19-iCre mice

**Fig 3:**
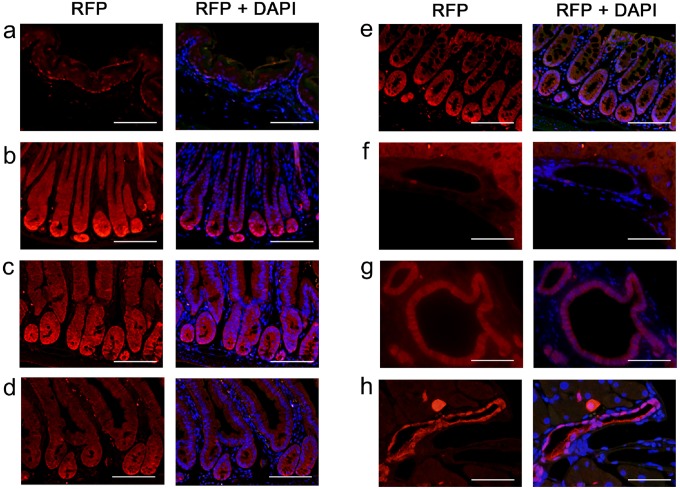
Expression patterns of Tomato (RFP) in the GI tract, hepatobiliary tissues, and the pancreas of adult Krt19-iCre; Tomato ^flox/+^ mice. Immunofluorescence of the epithelium and glands of the esophagus (**a**), fundus and pylorus of the stomach (**b**), duodenum (**c**), small intestine (**d**), colon (**e**), cholangiocytes of the intrahepatic bile duct and common bile duct (**f** and **g**, respectively), epithelial cells of the pancreatic duct and in some exocrine cells (**h**). Red: Tomato (RFP), Green: background (autofluorescence), Blue: DAPI. Scale bar: 50 μm.

To determine whether the reporter protein expression correlated with *iCre* mRNA expression in the GI tract, hepatobiliary tissues, and the pancreas, IF staining was performed with RFP antibody in tissue sections from the *Krt19-iCre*;*Tomato*^flox/+^ (*Krt19-iCre* Tomato reporter) mice (n = 3). Of all the samples, those of the GI tract, hepatobiliary tissues, and pancreas had RFP expression (Fig 3a–3h) corresponding to *iCre* mRNA expression determined by ISH (Fig 2). Further, biliary tree duct cells showed lower RFP expression levels than the epithelial cells of the GI tract (Fig 3a–3h), which is generally consistent with *iCre* mRNA expression in these cells (Fig 2). Esophageal epithelia and hepatocytes were labeled with anti-RFP antibody, however, these signals were also found in the negative control slides using rabbit IgG (Fig 3a and 3f, and S4 Fig). Moreover, some RFP-positive cells in the esophageal connective tissue were also labeled in the negative control. Although the unexpected mRNA expression of *iCre* among connective tissue cells of the esophagus was detected (Fig 2), the protein expression of iCre was uncommon. We confirmed the co-localization of RFP and endogenous Krt19 protein in the *Krt19-iCre* Tomato reporter mice (S5 Fig).

**Fig 4:**
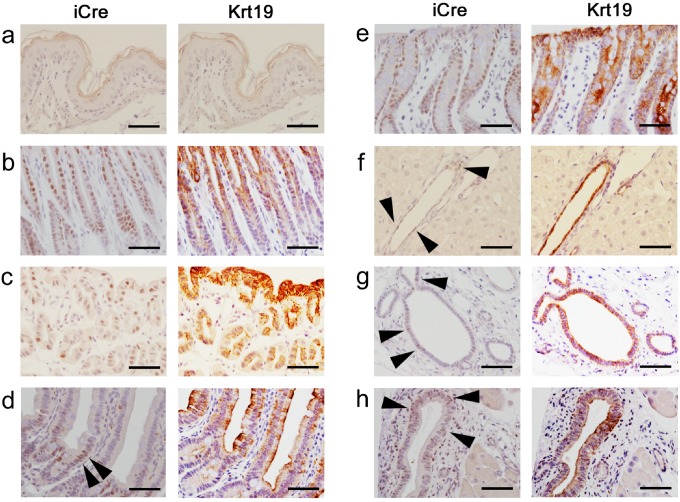
Expression patterns of iCre and Krt19 protein in the GI tract, hepatobiliary tissues, and the pancreas of adult Krt19-iCre; Tomato ^flox/+^mice. Immunohistochemistry of the esophageal epithelium (**a**), fundus and pylorus of the stomach (**b**), duodenum (**c**), small intestine (**d**), colon (**e**), cholangiocytes of the intrahepatic bile duct and common bile duct (**f** and **g**, respectively), and epithelial cells of the pancreatic duct (**h**) using serial sections. Scale bar: 50 μm.

To verify that iCre expression pattern corresponded to endogenous Krt19 expression, we performed immunohistochemistry for Krt19 and iCre (using anti-Cre antibody) on tissues from the GI tract, hepatobiliary organs, and pancreas using serial sections from *Krt19-iCre*-Tomato reporter mice (n = 3). The KRT19 and iCre protein expression patterns were consistent with each other in the epithelial cells of the stomach and colon (Fig 4b, 4c and 4e). Similarly, expression of endogenous KRT19 protein corresponded to that of iCre protein in the hepatobiliary and pancreatic ducts, as well as the stomach and colon (Fig 4f–4h). In the small intestine, iCre protein expression in the three samples was found mainly in transient amplifying cells and it was restricted compared to the endogenous Krt19 expression (Fig 4d). Though iCre expression was somewhat inconsistently correlated with endogenous Krt19 expression, reporter RFP protein expression patterns were observed over the entire intestinal epithelium, including the epithelial cells of the villi (Fig 3d and S5 Fig). Thus, we consider the *Krt19-iCre* mouse line to be a useful model to investigate GI tract epithelium, including the small intestine.

#### iCre expression in developmental stages of Krt19-iCre mice

**Fig 5:**
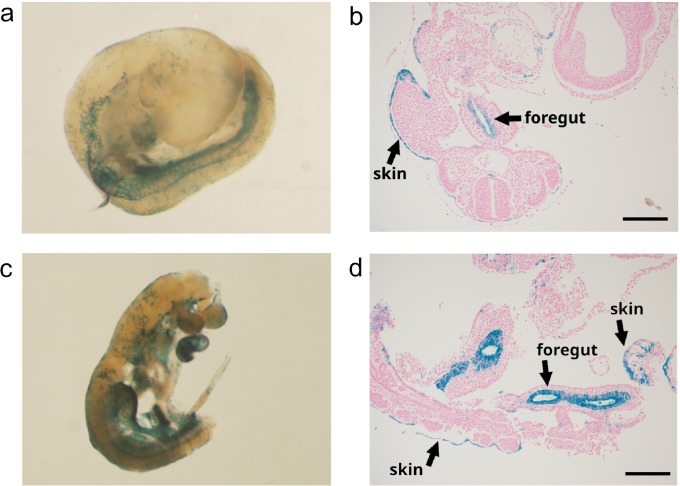
X-gal staining of E9.5 embryos from Krt19-iCre; LSL-LacZ ^flox/+^ mice. Whole-mount X-gal staining of two E9.5 embryos **(a and c)**, and its sagittal section (**b and d**) showing β-gal activity in the foregut and part of the skin in each embryo. Scale bars: 200 μm.

In order to characterize Krt19-iCre reporter expression during development, embryos resulting from *Krt19-iCre*×homozygous *LacZ* ^flox/flox^ mating were analyzed at different developmental stages. Embryos at E9.5, E12, and E17 stages, and newborn pups (P0) were obtained. The whole-mount X-gal staining of E9.5 embryos showed β-gal activity in the epithelium of the foregut and part of the skin (Fig 5a–5d). The β-gal activity in *Krt19-iCre; lacZ* embryos (E9.5) was equivalent to that of endogenous Krt19 previously reported in *Krt19* knock-in LacZ reporter mice [26]. In contrast, a previous report demonstrated that some E9.5 embryos of *Krt19-Cre; lacZ* mice expressed β-gal activity in the whole body, a wider tissue range than the expression of endogenous Krt19 [27]. The results indicate that *Krt19-iCre*micemight be a better model than the previous *Krt19-Cre* strain for studying early fetal development.

**Fig 6:**
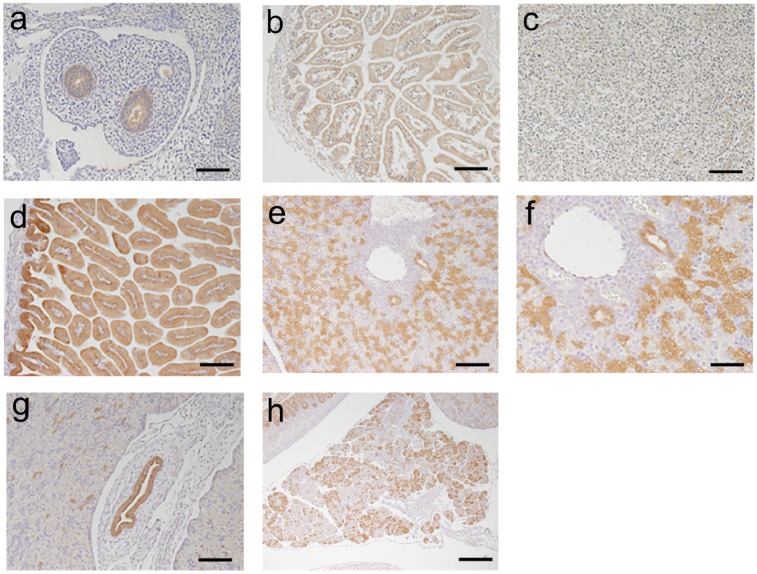
Expression patterns of β-gal activity in the GI tract, hepatobiliary tissues, and the pancreas ofKrt19-iCre; LSL-LacZ ^flox/+^ embryos and postnatal mice. Immunohistochemical detection of β-gal showed positive results in the epithelial cells of the GI tract in E12 (**a**) and E17 embryos (**b**). Very weak expression was detected in the liver, even in a E17 embryo (**c**). β-Gal expression was found in the GI tract (**d**), hepatobiliary tissues (**e**, **f**), pancreatic duct (**g**), and exocrine cells (**h**) of a postnatal P0 mouse. β-Gal-positive cells were distributed among both cholangiocytes and hepatocytes (**f**). Scale bars: 50 μm (**a-e**, **h**, **i**) and 25 μm (**f**).

An anti-β-galactosidase antibody for IHC was used to measure the expression level of the β-gal reporter at the cellular level. At E12, β-galactosidase was expressed in the glandular epithelial cells of the GI tract (Fig 6a). This expression was also shown at E17 and P0 (Fig 6b and 6d). Unlike in adult mice, iCre reporter expression was undetectable in the fetal liver at E17(Fig 6c). At P0, the expression pattern of β-galactosidase in cholangiocytes was similar to that of Krt19 in the intrahepatic duct of adult reporter mice (Fig 6e–6g). The expression of β-galactosidase was also detected in the epithelial cells of the biliary tree and in some epithelial cells of the exocrine glands in the pancreas in P0 mice (Fig 6h). The expression patterns of β-galactosidase at various stages of fetal development indicate that the Krt19-iCre mouse model is capable of tracing endogenous Krt19 expression in the GI tract, hepatobiliary tissues, and the pancreas during mouse development.

We also confirmed iCre expression in aged Krt19-iCre mice by IHC. The expression level of iCre protein was maintained in the stomach, small intestine, and colon. However, iCre expression in the hepatobiliary duct and pancreatic duct of Krt19-iCre mice at 18 months of age was weak compared to that in mice at eight weeks of age (S6 Fig).

#### KRT19 expression in the GI tract of human tissue

**Fig 7:**
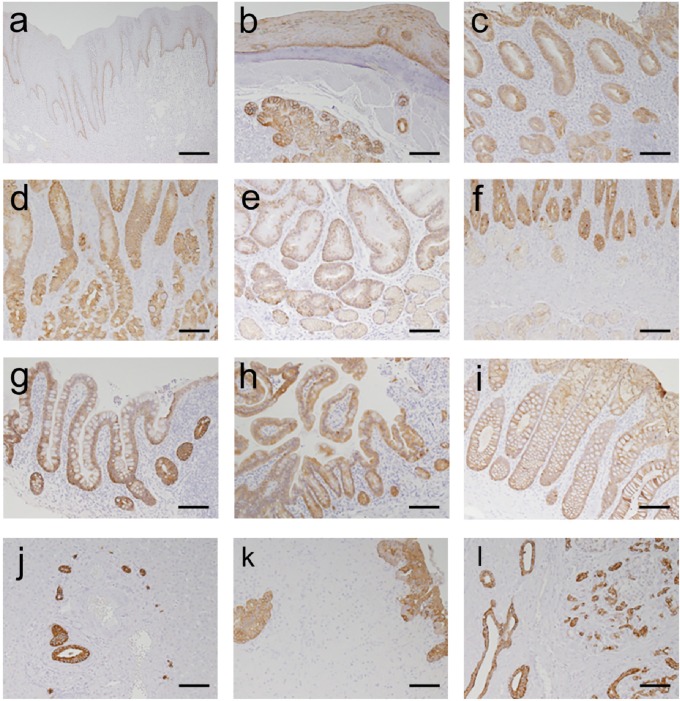
Expression pattern of KRT19 in the human GI tract, hepatobiliary tissues, and pancreas. Immunohistochemical detection of KRT19 showed positive results in the epithelial cells and exocrine gland cells of the oral mucosa (**a**), esophagus (**b**), fundus, gastric, and pyloric glands of stomach (**c-e**, respectively), duodenum(**f**), ileum(**g**), jejunum(**h**), and colon(**i**). The expression of KRT19 is also demonstrated in cholangiocytes of the intrahepatic and common bile ducts (**j**, **k**) and in epithelial cells of the pancreatic duct and in some exocrine cells (**l**). Scale bar: 100 μm.

To evaluate KRT19 expression in human tissues, we performed IHC with an anti-KRT19 antibody in normal tissue sections from the GI tract, hepatobiliary tissues, and the pancreas. KRT19 was expressed in the epithelial cells of the GI tract and the biliary tree, i.e., oral mucosa, esophagus (epithelial cells and glands), fundus and pylorus of the stomach, duodenum, ileum, jejunum, and colon(Fig 7). These results demonstrate that the expression of KRT19 in humans closely agreed with that in Krt19-iCre Tomato reporter mice (Fig 3). In human tissues, epithelial cells of the biliary tree had similar KRT19 expression levels as those of the GI tract (Fig 7c–7l). However, in Krt19-iCre Tomato reporter mice, KRT19 expression levels were lower in the epithelial cells of the biliary tree than in the GI tract (Fig 3g and 3h). In a previous report, Krt19-CreERT mice showed similar Krt19 reporter expression patterns in the biliary tree [8]. Taken together, the lower reporter expression in the epithelial cells of the biliary tree may represent mouse Krt19 promoter-dependence rather than an actual difference between Cre and iCre-dependent expressions.

## Conclusion

The expression of iCre recombinase from the *Krt19* locus in adult mice was apparent in the epithelial cells in the GI tract, intrahepatic and common bile duct, and the pancreatic duct.

We suggest that the *Krt19-iCre* mouse line we established might be a better model than the current models (*Krt19-Cre* and *Krt19-CreERT* lines) for the following two reasons: First, the β-galactosidase activity in the *Krt19-iCre; lacZ* embryos (E9.5) was equivalent to that of endogenous Krt19previously reported in *Krt19* knock-in LacZ reporter mice. Second, this is the first model to demonstrate the expression patterns of Cre recombinase using β-gal at various fetal and neonatal stages. The Cre expression patterns during later fetal and neonatal stages in the current mouse line models for Krt19, Krt19-Cre and Krt19-CreERT were not analyzed. The Krt19-iCre line we established is more suitable for use in Cre-mediated DNA recombination mouse models for embryological study.

Together, we conclude that the generated *Krt19-iCre* transgenic mouse line is a valid and useful tool for studying most stages, including fetal and neonatal, of epithelial cell development. In the comparison with human samples in this study, the expression of iCre in this transgenic mouse line generally agreed with the expression pattern of KRT19 in human organs. Thus, genomic editing using the iCre-loxP system, more efficient than the traditional Cre-loxP system, may enable the analysis of pathological conditions.

## Supporting information

S1 Fig RT-PCR of iCre RNA in the GI tract, hepatobiliary tissues, and the pancreas of adult Krt19-iCre mice. Quantitative analysis of *iCre* mRNA from tissues of Krt19-iCre (iCre) mice and wild-type (WT) mice (n = 3 each). Stomach (a), duodenum (b), small intestine (c), colon (d), intrahepatic bile duct with liver (e), common bile duct (f), and pancreas (g). Bars represent the average of triplicate measured values, which were normalized to β-actin.
(EPS)

S2 Fig Expression of *iCre* RNA and endogenous Krt19 protein in the GI tract, hepatobiliary tissues, and the pancreas of adult Krt19-iCre mice. Combined ISH of iCre and IF of Krt19 to confirm the co-localization of iCre RNA expression and endogenous Krt19 protein. Representative photos of stomach epithelium (**a**), duodenum (**b**), small intestine (**c**), colon (**d**), cholangiocytes of the intrahepatic bile duct and common bile duct (**e** and **f**, respectively), and epithelial cells of the pancreatic duct(**g**). Scale bars: 50μm.
(EPS)

S3 Fig RNA expressions by negative control probe in the GI tract, hepatobiliary tissues, and the pancreas of adult Krt19-iCre mice. Representative photos of the esophageal epithelium (**a**), stomach (**b**), duodenum (**c**), small intestine (**d**), colon (**e**), cholangiocytes of the intrahepatic bile duct and common bile duct (**f** and **g**, respectively), and epithelial cells of the pancreatic duct and in some exocrine cells (**h**). Scale bars: 50μm.
(EPS)

S4 Fig Immunohistochemistry for negative control rabbit IgG labeled with RFP. Immunohistochemistry of esophageal epithelia (a) and hepatocytes (b) for negative control rabbit IgG labeled with RFP. Scale bars: 50μm.
(EPS)

S5 Fig Expression of RFP and endogenous Krt19 protein in the GI tract, hepatobiliary tissues, and the pancreas of adult Krt19-iCre; Tomato^flox/+^ mice. Dual IF of RFP and Krt19 to confirm the co-localization of reporter protein RFP expression and endogenous Krt19 protein. Representative photos of stomach epithelium (**a**), duodenum (**b**), small intestine (**c**), colon (**d**), cholangiocytes of the intrahepatic bile duct and common bile duct (**e** and **f**, respectively), and epithelial cells of the pancreatic duct(**g**). Scale bars: 50μm.
(EPS)

S6 Fig Expression of iCre protein in the GI tract, hepatobiliary tissues, and the pancreas of P450 Krt19-iCre; Tomato ^flox/+^ mice. Immunohistochemistry of stomach epithelium (a), small intestine (b), colon (c), cholangiocytes of the intrahepatic bile duct and common bile duct (d and e, respectively), and epithelial cells of the pancreatic duct (f). Scale bars: 50 μm.
(EPS)

S1 Table Characteristics of patients and tissues shown in Fig 7. (EPS)

## References

[1] Barak V, Goike H, Panaretakis KW, Einarsson R. Clinical utility of cytokeratins as tumor markers. Clin Biochem. 2004;37(7):529–40. 10.1016/j.clinbiochem.2004.05.009 . 15234234

[2] Brembeck FH, Moffett J, Wang TC, Rustgi AK. The keratin 19 promoter is potent for cell-specific targeting of genes in transgenic mice. Gastroenterology. 2001;120(7):1720–8. 10.1053/gast.2001.24846 . 11375953

[3] Asfaha S, Hayakawa Y, Muley A, Stokes S, Graham TA, Ericksen RE, et al Krt19(+)/Lgr5(-) Cells Are Radioresistant Cancer-Initiating Stem Cells in the Colon and Intestine. Cell Stem Cell. 2015;16(6):627–38. 10.1016/j.stem.2015.04.013 . 26046762 PMC4457942

[4] Chu PG, Weiss LM. Keratin expression in human tissues and neoplasms. Histopathology. 2002;40(5):403–39. . 12010363 10.1046/j.1365-2559.2002.01387.x

[5] Jain R, Fischer S, Serra S, Chetty R. The use of Cytokeratin 19 (CK19) immunohistochemistry in lesions of the pancreas, gastrointestinal tract, and liver. Appl Immunohistochem Mol Morphol. 2010;18(1):9–15. 10.1097/PAI.0b013e3181ad36ea . 19956064

[6] Harada N, Tamai Y, Ishikawa T, Sauer B, Takaku K, Oshima M, et al Intestinal polyposis in mice with a dominant stable mutation of the beta-catenin gene. EMBO J. 1999;18(21):5931–42. 10.1093/emboj/18.21.5931 . 10545105 PMC1171659

[7] Zhao GF, Zhao S, Liu JJ, Wu JC, He HY, Ding XQ, et al Cytokeratin 19 promoter directs the expression of Cre recombinase in various epithelia of transgenic mice. Oncotarget. 2017;8(11):18303–11. Epub 2017/04/15. 10.18632/oncotarget.15435 . 28407687 PMC5392329

[8] Means AL, Xu Y, Zhao A, Ray KC, Gu G. A CK19(CreERT) knockin mouse line allows for conditional DNA recombination in epithelial cells in multiple endodermal organs. Genesis. 2008;46(6):318–23. 10.1002/dvg.20397 . 18543299 PMC3735352

[9] Shimshek DR, Kim J, Hubner MR, Spergel DJ, Buchholz F, Casanova E, et al Codon-improved Cre recombinase (iCre) expression in the mouse. Genesis. 2002;32(1):19–26. . 11835670 10.1002/gene.10023

[10] Bridges PJ, Koo Y, Kang DW, Hudgins-Spivey S, Lan ZJ, Xu X, et al Generation of Cyp17iCre transgenic mice and their application to conditionally delete estrogen receptor alpha (Esr1) from the ovary and testis. Genesis. 2008;46(9):499–505. 10.1002/dvg.20428 . 18781648 PMC2637183

[11] Cacioppo JA, Koo Y, Lin PC, Gal A, Ko C. Generation and characterization of an endothelin-2 iCre mouse. Genesis. 2015;53(2):245–56. 10.1002/dvg.22845 . 25604013 PMC4382364

[12] Cacioppo JA, Koo Y, Lin PC, Osmulski SA, Ko CD, Ko C. Generation of an estrogen receptor beta-iCre knock-in mouse. Genesis. 2016;54(1):38–52. 10.1002/dvg.22911 . 26663382 PMC4756916

[13] Park CJ, Chen G, Koo Y, Lin PP, Cacioppo JA, Prohaska H, et al Generation and characterization of an estrogen receptor alpha-iCre knock-in mouse. Genesis. 2017 10.1002/dvg.23084 . 29115049 PMC5951291

[14] Lee EC, Yu D, Martinez de Velasco J, Tessarollo L, Swing DA, Court DL, et al A highly efficient Escherichia coli-based chromosome engineering system adapted for recombinogenic targeting and subcloning of BAC DNA. Genomics. 2001;73(1):56–65. 10.1006/geno.2000.6451 . 11352566

[15] Liu P, Jenkins NA, Copeland NG. A highly efficient recombineering-based method for generating conditional knockout mutations. Genome Res. 2003;13(3):476–84. 10.1101/gr.749203 . 12618378 PMC430283

[16] Testa G, Zhang Y, Vintersten K, Benes V, Pijnappel WW, Chambers I, et al Engineering the mouse genome with bacterial artificial chromosomes to create multipurpose alleles. Nat Biotechnol. 2003;21(4):443–7. 10.1038/nbt804 . 12627172

[17] Warming S, Costantino N, Court DL, Jenkins NA, Copeland NG. Simple and highly efficient BAC recombineering using galK selection. Nucleic Acids Res. 2005;33(4):e36 10.1093/nar/gni035 . 15731329 PMC549575

[18] Thomason LC, Sawitzke JA, Li X, Costantino N, Court DL. Recombineering: genetic engineering in bacteria using homologous recombination. Curr Protoc Mol Biol. 2014;106:1 16 1–39. 10.1002/0471142727.mb0116s106 . 24733238

[19] Gebhard S, Hattori T, Bauer E, Bosl MR, Schlund B, Poschl E, et al BAC constructs in transgenic reporter mouse lines control efficient and specific LacZ expression in hypertrophic chondrocytes under the complete Col10a1 promoter. Histochem Cell Biol. 2007;127(2):183–94. 10.1007/s00418-006-0236-8 . 17051351 PMC1779629

[20] Ting JT, Feng G. Recombineering strategies for developing next generation BAC transgenic tools for optogenetics and beyond. Front Behav Neurosci. 2014;8:111 10.3389/fnbeh.2014.00111 . 24772073 PMC3982106

[21] Chandler KJ, Chandler RL, Broeckelmann EM, Hou Y, Southard-Smith EM, Mortlock DP. Relevance of BAC transgene copy number in mice: transgene copy number variation across multiple transgenic lines and correlations with transgene integrity and expression. Mamm Genome. 2007;18(10):693–708. Epub 2007/09/21. 10.1007/s00335-007-9056-y . 17882484 PMC3110064

[22] Madisen L, Zwingman TA, Sunkin SM, Oh SW, Zariwala HA, Gu H, et al A robust and high-throughput Cre reporting and characterization system for the whole mouse brain. Nat Neurosci. 2010;13(1):133–40. 10.1038/nn.2467 . 20023653 PMC2840225

[23] Soriano P. Generalized lacZ expression with the ROSA26 Cre reporter strain. Nat Genet. 1999;21(1):70–1. 10.1038/5007 . 9916792

[24] Wang F, Flanagan J, Su N, Wang LC, Bui S, Nielson A, et al RNAscope: a novel in situ RNA analysis platform for formalin-fixed, paraffin-embedded tissues. J Mol Diagn. 2012;14(1):22–9. 10.1016/j.jmoldx.2011.08.002 . 22166544 PMC3338343

[25] Stempel AJ, Morgans CW, Stout JT, Appukuttan B. Simultaneous visualization and cell-specific confirmation of RNA and protein in the mouse retina. Mol Vis. 2014;20:1366–73. Epub 2014/10/30. . 25352743 PMC4169891

[26] Tamai Y, Ishikawa T, Bosl MR, Mori M, Nozaki M, Baribault H, et al Cytokeratins 8 and 19 in the mouse placental development. J Cell Biol. 2000;151(3):563–72. 10.1083/jcb.151.3.563 . 11062258 PMC2185583

[27] Means AL, Chytil A, Moses HL, Coffey RJ Jr., Wright CV, Taketo MM, et al Keratin 19 gene drives Cre recombinase expression throughout the early postimplantation mouse embryo. Genesis. 2005;42(1):23–7. 10.1002/gene.20119 . 15828001

